# Factors Associated With the Rate of Recovery After Cervical Decompression Surgery for Degenerative Cervical Myelopathy: A Retrospective Analysis

**DOI:** 10.7759/cureus.39654

**Published:** 2023-05-29

**Authors:** Anthony N Baumann, Mingda Chen, Peter Ahorukomeye, Christopher G Furey, Christina W Cheng

**Affiliations:** 1 Department of Rehabilitation Services, University Hospitals Cleveland Medical Center, Cleveland, USA; 2 School of Medicine, Case Western Reserve University, Cleveland, USA; 3 Department of Orthopedic Surgery, University Hospitals Cleveland Medical Center, Cleveland, USA

**Keywords:** strength, balance, numbness, cervical spine surgery, degenerative cervical myelopathy

## Abstract

Introduction

Degenerative cervical myelopathy (DCM) is a debilitating spinal condition with a wide variety of symptoms that can differ greatly among individuals. Common symptoms include numbness, extremity weakness, loss of balance, and gait instability. Decompression surgeries are commonly indicated for the treatment of DCM with varying outcomes reported in the literature. However, there is little evidence on the rate of recovery defined as the time until improvement in symptoms such as numbness, balance, and strength after surgery for DCM. The purpose of this study was to determine the rate of neurological recovery after surgery for DCM and its subsequent association with various risk factors to guide clinicians while providing care and improve patient education.

Methods

This study was a retrospective case series (n=180 patients) examining patients who underwent cervical decompression surgery for DCM. All patients had a clinical presentation of DCM, were diagnosed with DCM, had radiographic degenerative changes and cervical stenosis, and received surgical management from 2010 to 2020 in a tertiary hospital system. Data recorded included age, smoking status, duration of pre-operative symptoms, preoperative and postoperative pain, and postoperative rate of recovery (days until improvement) in numbness, upper extremity strength, and balance.

Results

Patients (n=180) had an average age of 65.7 years (SD ±9.2 years, range 43-93 years). The mean ± standard deviation for the rate of recovery (days until improvement) in numbness, upper extremity strength, and balance was 84.5 ± 94.4 days, 50.6 ± 42.8 days, and 60.4 ± 69.9 days, respectively. There was only a marginally significant association between the rate of recovery for numbness after surgery and patient age (p=0.053). The average rate of recovery in numbness for patients older than 60 years was significantly longer than those younger than 60 years (99.3 versus 60.2 days). Preoperative smoking status was significantly associated with persistent moderate to severe pain (p=0.032) within the six-month postoperative period. No significant correlations were seen between the rate of recovery for balance or strength and patient age or preoperative duration of symptoms.

Conclusion

There was great variability in the rate of recovery for postoperative symptoms after surgery for DCM. A longer time for improvement in postoperative numbness was only marginally correlated with the increased patient age after surgery for DCM. There was no correlation found between strength or balance recovery times and patient age. Smoking status was associated with moderate to severe postoperative pain after surgery for DCM. Furthermore, the duration of preoperative symptoms was not associated with improvement in postoperative symptoms after surgery for DCM. More research is needed to determine factors impacting the rate of recovery after surgery for DCM.

## Introduction

Degenerative cervical myelopathy (DCM) is a common, progressive spinal disorder with a wide variety of symptoms due to degenerative changes in the cervical spine, which result in spinal cord compression [1-3]. The implication of this disease is immense as DCM is one of the most common causes of spinal cord dysfunction in older adults [1,2]. Furthermore, symptoms of DCM can occur gradually, and can vary among individuals [2-5]. Symptoms of DCM include loss of fine motor control and strength in the upper and lower extremities, neck pain and/or upper extremity pain, numbness in the upper extremities and/or lower extremities, and impairment in gait and balance [2-3]. Cervical decompression surgery is the only treatment that can definitively stop the progression of the disease and potentially improve function [1,2,6-8]. Although significant emphasis is placed on preventing the progression of disease with surgical decompression, there is limited data that exists regarding the rate of recovery for subjective and/or objective symptoms in relation to patient-specific factors, such as age and duration of preoperative symptoms [3].

In contrast to the limited evidence on subjective symptom improvement after decompression surgery for DCM, there are numerous studies in the literature that have examined objective outcomes that are not easily understood by a patient [1,3]. For example, decompression surgery for DCM has been shown to improve scores on the Neck Disability Index (NDI) as well as improve cervical alignment [1,6,9]. While one study examined qualitative improvements in gait mechanics in patients after surgery for DCM, other studies in the literature have focused on quantitative assessments of gait via patient outcome tools that can be difficult to understand for a patient [3]. As another example, some studies reported that factors such as age, ambulatory status, and smoking status have been shown to predict both imaging outcomes and Japanese Orthopedic Association (JOA) scores in patients after surgery for DCM [10,11]. These outcomes are relevant to patient care, but not easily understood by the patient during preoperative or postoperative counseling. The lack of easily understood outcomes has the ability to impact patient education and damage the patient-physician relationship.

Despite the existing literature on postoperative outcomes such as subjective symptoms and strength improvement after cervical decompression for DCM, no study to date has examined the rate of recovery, defined as the time until symptomatic improvement in muscle strength, numbness, and balance status, in a single cohort to determine the risk factors predisposing patients to prolonged recovery after surgery for DCM [1,2]. The findings of this study can help guide surgeons when counseling their patients about the progression of their recovery in order to manage patient expectations, improve patient satisfaction, and improve outcomes by facilitating communication between the patient and the physician. The purpose of this study was to quantify rate of recovery in several subjective and objective symptoms commonly seen in DCM (numbness, motor function, and balance) as well as correlate these symptoms with patient age, comorbidities, smoking status, and duration of preoperative symptoms to help provide an objective time frame for the rate of recovery as to when patients can begin to expect improvement in their symptoms after surgery for DCM.

## Materials and methods

Study design

This study was a retrospective case series of 180 patients who underwent cervical decompression for DCM with radiographic evidence of cervical stenosis between 2010 and 2020 in a single hospital system. All patients were treated at a single tertiary referral center with surgeries performed by both orthopedic surgeons and neurosurgeons. The current study was approved by the University Hospitals Cleveland Medical Center Institutional Review Board (study number 20211740).

Inclusion and exclusion criteria

Patients older than 18 years, with a diagnosis of cervical myelopathy in the physician’s note, radiographic evidence of cervical spine degenerative changes, International Classification of Diseases, 10th Revision (ICD-10) code for cervical stenosis (M48.02), and cervical decompression surgery for DCM were included. Exclusion criteria were spinal cord compression due to cancer or trauma and age less than 18 years. Patients with a history of cervical stenosis and subsequent surgical correction to the cervical spine were selected from patient charts received from the University Hospitals Cleveland Medical Center.

Study definitions

As defined in this study, DCM refers to cervical spinal cord compression caused by age-related degenerative and stenotic changes in the cervical spine. Cervical decompression surgery, as mentioned in this study, includes surgical procedures such as cervical laminectomy with or without single- or multi-level cervical discectomy and fusion, and cervical corpectomy. Decompression surgeries were completed via an anterior or posterior approach. For the purpose of this study, the rate of recovery was defined as the time until initial improvement (days) in the patient's symptoms (numbness, upper extremity strength, or balance). The rate of recovery reflects when the patient or the physician first reported improvement of any magnitude in symptoms and does not reflect symptom abolishment. Improvement in balance status was determined by a patient’s subjective reporting of improved balance and/or ability to ambulate. Improvement in numbness was determined by the patient subjective report. Improvement in strength was determined by the patient subjective report or by physician testing per manual muscle testing. The rate of recovery data was only included in each symptom data set if the patient showed improvement in the symptom category due to the need for numerical values for measurement. No recovery was recorded if the patient did not demonstrate improvement or if no information was recorded for the symptom category.

Data collection

Data recorded included patient age (years), smoking status (current, previous, never), duration of preoperative symptoms (days), postoperative pain (0-10 Visual Analog Scale), diabetic status, and the rate of recovery in postoperative outcomes of numbness, balance, and strength (days). The primary outcomes recorded were the number of days until the first documented improvement in numbness, upper extremity motor strength, and balance. Using available information in the electronic medical records, the majority of patients were followed up for at least one year after their surgery for DCM.

Statistical analysis

The means and standard deviations were calculated for continuous variables and frequencies of categorical variables where appropriate. Statistical analysis was performed to evaluate the association between a continuous outcome variable and a categorical exposure variable using the two-sample t-test if there were two categories, or by one-way ANOVA if there were more than two categories.

## Results

Patients (n=180) in our cohort had an average age of 65.7 years (SD ±9.2 years, range 43-93 years). In terms of risk factors and comorbidities, 38 patients (21.2%) were current smokers, 53 patients (29.6%) were smokers in the past but did not currently smoke, and 43 patients (23.9%) had diabetes at the time of surgery. The average duration of preoperative symptoms in patients with DCM was 625.9 ± 982.0 days. For the primary study outcomes, the mean rate of recovery was 50.6 ± 42.7 days for upper extremity motor strength, 60.4 ± 69.9 days for balance, and 84.5 ± 94.4 for upper extremity numbness. For the study outcome of rate of recovery in numbness, 65.5% of patients (n=118) had recorded improvement after surgery for DCM. For the study outcome of rate of recovery in upper extremity strength, 56.1% of patients (n=101) had recorded improvement after surgery for DCM. For the study outcome of rate of recovery in balance, 63.3% of patients (n=114) recorded improvement after surgery for DCM. The rate of recovery for numbness (range 6-417 days), upper extremity strength (range 6-216 days), and balance (range 12-377 days) after surgery for DCM was highly variable among individual patients. Table 1 provides more information on patient demographics and the time until improvement in symptoms after surgery for DCM.

**Table 1:** Patient demographics and rate of recovery in postoperative symptoms

Patient demographics and symptom categories	Number of patients (n=180)	Mean ± standard deviation	Median	Range
Age (years)		65.7 ± 9.2	65	43-93
Smoking (%)	179 (99.4%)			
Never	88 (49.2%)			
Previous	53 (29.6%)			
Current	38 (21.2%)			
Diabetes (%)	180 (100.0%)			
No	137 (76.1%)			
Yes	43 (23.9%)			
Duration of preoperative symptoms (days)	140 (77.8%)	625.9 ± 982.0	270.0	10-6840
Postoperative rate of recovery in numbness (days)	118 (65.6%)	84.5 ± 94.4	40.5	6-417
Postoperative rate of recovery in upper extremity motor function (days)	101 (56.1%)	50.6 ± 42.8	36.0	6-216
Postoperative rate of recovery in balance (days)	114 (63.3%)	60.4 ± 69.9	32.5	12-377

There was a marginally significant association between the rate of recovery in numbness after surgery for DCM and patient age (p=0.053). The average time until improvement in numbness for patients older than 60 years was significantly longer than for those younger than 60 years (99.3 days in the older cohort versus 60.2 days in the younger cohort). Additionally, the preoperative smoking status was significantly associated with persistent moderate to severe pain (defined as 7/10 or greater on the pain scale) within the six-month postoperative period (p=0.032). No significant association was found between postoperative outcomes for the rate of recovery in numbness, strength, or balance and preoperative duration of symptoms, smoking status, and diabetic status. Table 2 provides more information on associations between postoperative symptoms and patient-specific factors.

**Table 2:** Association between postoperative rate of recovery and preoperative patient characteristics (age at first surgery, smoking, diabetes, duration of preoperative symptoms)

Postoperative outcome	Age at surgery (years)			Smoking				Diabetes			Duration of preoperative symptoms			
	≤60	>60	p-value	Never	Previous	Current	p-value	No	Yes	p-value	≤6 months		>6 months	p-value
Patients (n)	50	130		88	53	38		137	43		58		82	
Rate of recovery in numbness (days)	60.2 ± 76.5	93.9 ± 99.3	0.053	95.6 ± 110.2	81.2 ± 86.2	65.0 ± 62.4	0.281	87.9 ± 95.8	69.4 ± 88.8	0.391	93.3 ± 105.4		72.2 ± 74.2	0.289
Rate of recovery in upper extremity strength (days)	56.9 ± 45.7	47.6 ± 41.3	0.328	48.2 ± 35.1	49.0 ± 42.4	57.3 ± 56.1	0.772	52.7 ± 45.8	43.1 ± 29.0	0.239	45.8 ± 40.4		51.8 ± 41.8	0.518
															Rate of recovery in balance (days)	67.8 ± 84.5	57.8 ± 64.3	0.555	65.1 ± 76.2	64.4 ± 76.2	43.9 ± 35.4	0.174	60.7 ± 67.3	59.3 ± 81.3	0.943	64.0 ± 82.6		55.1 ± 61.1	0.575
																														Presence of persistent moderate-severe pain within 6 months after surgery (days)	1.7 ± 3.0	0.6 ± 1.5	0.15	0.8 ± 1.9	0.2 ± 0.5	1.8 ± 2.9	0.032	0.9 ± 2.1	1.3 ± 2.5	0.649	0.9 ± 2.1		1.3 ± 2.5	0.649

## Discussion

DCM is a debilitating spinal condition that can present with a wide variation in symptomology and rate of progression [1-3,5]. The current study retrospectively examined the rate of recovery for common subjective and objective symptoms of DCM as well as factors associated with delayed improvement after surgical decompression intervention for DCM to better understand patient outcomes and provide information that can aid in preoperative and postoperative patient counseling. Many of the studies in the literature examine objective outcomes that are not easily understood by patients in the clinic, such as JOA scores [6,9-12]. Other studies in the literature have examined subjective symptoms, such as preoperative and postoperative pain and numbness, but did not measure changes in strength or balance outcomes in a single cohort [13]. There is also a realization in the literature that there is a need to provide quantitative data on subjective outcomes, such as gait, in ways that cannot be assessed with patient outcome tools [3]. Therefore, the current study highlights a gap in the literature by examining the rate of recovery for numbness, strength, and balance in a single cohort while examining the correlation between time to improvement for symptoms and relevant patient-specific factors.

Overall, the rate of recovery for postoperative numbness, balance, and strength after decompression surgery for DCM was not significantly correlated with patient-specific factors such as age, duration of preoperative symptoms, diabetic status, and smoking status. In the current study, a longer time to improvement in postoperative numbness was only marginally correlated with increased patient age after surgery for DCM. Based on the results of the current study, younger patients (<60 years old) could have a quicker rate of recovery in numbness after surgery for DCM as compared to older patients (>60 years old) (p=0.053). The current study found no correlation between strength or balance improvement times and patient-specific factors, indicating similar improvement in symptoms among patients with different ages, duration of symptoms, and comorbidities. On average, the rate of recovery in symptoms of numbness, upper extremity strength, and balance was about two to three months. These findings are important as other studies in the literature have examined ways to quantify subjective symptoms [3].

In the current study, higher levels of pain after decompression surgery for DCM were seen in patients who smoked prior to surgery. This finding agrees with the literature as higher levels of pain and alterations in bone healing have been seen after surgery in patients who smoke [14]. On a pathophysiological scale, cigarettes contain numerous toxins that could impair bone healing, even though the exact mechanism is elusive and not well-understood [14]. The literature suggests that smoking cessation about one month prior to surgical intervention can reduce complications [14]. As suggested in this study, higher levels of postoperative pain after decompression surgery for DCM could possibly be an avoided complication via smoking cessation, although more research is needed to determine a dose-response relationship. This information on symptomatic improvement can allow physicians to counsel patients preoperatively to help manage expectations after surgery, provide motivation for smoking cessation, and help improve patient satisfaction via the patient-physician relationship. If a patient believes that his or her symptoms will resolve spontaneously after surgery for DCM, a normal time frame of two to three months before seeing improvement could be discouraging, thus possibly limiting their recovery postoperatively and increasing patient dissatisfaction. Although it is well-documented that decompression surgeries can provide meaningful improvements in patient-recorded outcomes, limited data exists to determine the patient-specific rate of recovery in those symptoms [15]. While some studies have examined changes in patient strength after surgery, the literature does not report a specific quantitative time to improvement measure for strength in patients with DCM [2]. Rather, most studies focus on a pre-determined time to reassess symptoms, thus limiting information on the rate of the patient’s recovery after surgery for DCM. This study adds to the current literature on patient outcomes by providing a rate of recovery for subjective and objective symptoms readily experienced by and easily communicated to the patient.

Future research needs to focus on other relevant patient-specific factors, such as body mass index and obesity levels, that could impact the overall rate of recovery as well as degree of improvement in symptoms in relation to time. Unfortunately, the level of obesity was not recorded in this study and the relationship between the rate of recovery after decompression surgery for DCM and obesity remains unknown. Furthermore, different surgical factors, such as laminectomy with or without fusion or posterior versus anterior approach, may affect the rate of recovery and need to be elucidated in future studies. For the current study, the rate of recovery for numbness and balance via patient report was chosen as a good measure because numbness and balance are hard to objectively quantify in the clinic, but are important to the patient. On the other hand, the motor score is easily examined by the clinician through quick strength examination techniques, but these tests are limited in terms of sensitivity to improvement. The more information that can be available to the patient and the physician regarding postoperative recovery, the greater the potential for improvement in patient satisfaction, patient-physician relationship, and patient outcomes.

The current study has several limitations that can impact the application of the results. One limitation of this study is that the data relied largely on the reported improvement by the patient to the treating physician, which may or may not accurately describe when improvements were actually seen in the day-to-day life of the patient. The retrospective nature of this study and the reliance on medical documentation impair the results of this study. These limitations require that the conclusions of this study be taken cautiously; however, the findings of this study can spur future higher level research in this area. Further research can focus on prospective studies to determine the rate of improvement in symptoms after surgery for DCM. As noted above, the current study found that between 56% and 65% of patients reported improvement in numbness, balance, and upper extremity strength. Unfortunately, patients who did not follow up with their physician as regularly due to outliers in outcomes, such as immediate improvement or very poor health, would not have been consistently reflected in the data. A larger sample size from multiple health systems in a prospective study would help eliminate this study limitation. Also, if a patient did not show improvement in a symptom category, that data was not included as no quantitative measure could be created. Therefore, the data in this study only shows the rate of recovery in those patients who did improve after surgery for DCM. This study does not show the rate of recovery across all patients; if improvement was not documented for a patient, it is uncertain if no improvement was truly seen or if symptom improvement was simply not documented. Finally, this study did not account for some factors that could impact patient outcomes and reveal additional relationships between patient variables, such as anterior or posterior approach for cervical surgery or the number of patients with decompression alone versus decompression with fusion. More research is needed to determine the true rate of symptom recovery after surgery for DCM and its subsequent relationship with patients and surgical factors.

## Conclusions

There is great variability in the rate of recovery as defined by time until improvement in postoperative numbness, balance, and strength after cervical decompression surgery for DCM. Many patient-specific factors are not significantly associated with the rate of recovery, and the average rate of recovery for postoperative numbness, balance, and strength after surgery for DCM was two to three months. A longer time to improvement in postoperative numbness is only marginally associated with the increased patient age, but not the duration of preoperative symptoms, after surgery for DCM. No significant association between the rate of recovery in postoperative strength or balance and patient-specific factors, such as age, duration of symptoms, smoking status, and diabetes, was found in this study. However, history of smoking was significantly associated with moderate to severe postoperative pain after surgery for DCM. More research is needed to determine other patient-specific and surgical factors influencing the rate of symptom recovery after surgery for DCM to achieve effective patient-physician communication and improve patient satisfaction.
